# Case Report: Clinicopathological features of bladder neuroendocrine neoplasm: a retrospective case series of nine cases and literature review

**DOI:** 10.3389/fonc.2026.1841019

**Published:** 2026-08-05

**Authors:** Xinni Ye, Peng Chen, Junhao Chen, Zhongsong Zhang, Li Zhao, Yuanyin Teng, Peiqin Zhan, Xiangyun Li, Junxian Zhao, Lingxiang Wen, Zuqing Nie, Yuanzhi Fu, Jieming Zuo, Haihao Li, Zhaojiao Li, Jia Wang, Bo Chen, Haifeng Wang, Shi Fu

**Affiliations:** 1 Department of Urology, The Second Affiliated Hospital of Kunming Medical University, Kunming, China; 2 School of Clinical Medicine, Chengdu Medical College, Chengdu, Sichuan, China; 3 Department of Anesthesiology, The First Affiliated Hospital of Kunming Medical University, Kunming, Yunnan, China; 4 Institute of Hematology, Zhejiang University, Hangzhou, Zhejiang, China; 5 Department of Urology, 920th Hospital of Joint Logistics Support Force of Chinese People’s Liberation Army, Kunming, Yunnan, China; 6 School of Clinical Medicine, Kunming Medical University, Kunming, Yunnan, China; 7 Department of Central Lab, The Second Affiliated Hospital of Kunming Medical University, Kunming, Yunnan, China; 8 Department of Clinical Medicine, Kunming University of Science and Technology, Kunming, Yunnan, China; 9 Department of Neurology, Qujing Second People’s Hospital, Qujing, Yunnan, China; 10 Department of Endocrinology, The Second Affiliated Hospital of Kunming Medical University, Kunming, China; 11 Department of Urology, Qujing Second People’s Hospital, Qujing, Yunnan, China

**Keywords:** bladder neuroendocrine neoplasm, clinicopathological features, immunohistochemistry, immunotherapy, multimodal therapy

## Abstract

Primary bladder neuroendocrine neoplasm (BNEN) is an uncommon but clinically aggressive malignancy, and evidence guiding its diagnosis and management remains limited. This retrospective study analyzed nine patients diagnosed with BNEN at a single institution between 2014 and 2024, with pathological classification confirmed by morphology and neuroendocrine immunohistochemical markers. Clinical features, histological subtypes, treatment strategies, and follow-up outcomes were reviewed. Most patients presented with painless gross hematuria and advanced-stage disease. Histologically, the cohort showed marked heterogeneity, including small cell neuroendocrine carcinoma (SCNEC), large cell neuroendocrine carcinoma (LCNEC), paraganglioma (PGL), and mixed neuroendocrine neoplasms. All cases expressed CD56 and synaptophysin, while Ki-67 indices indicated high proliferative activity in most tumors. Despite multimodal treatment, prognosis remained poor. Findings related to immunotherapy were preliminary and should be interpreted with caution. These findings highlight the aggressive and heterogeneous nature of BNEN and support individualized multimodal management, with further studies needed to clarify the role of immunotherapy.

## Background

According to the 2022 cancer burden data released by the National Cancer Center of China, an estimated 92,900 new cases of bladder cancer and 41,400 deaths occurred in China in 2022. The crude incidence and mortality rates were 6.58/100,000 and 2.93/100,000, respectively, while the age-standardized incidence and mortality rates were 3.44/100,000 and 1.34/100,000, respectively (1). Primary bladder BNEN is a rare non-urothelial neoplasm of the bladder, accounting for less than 1% of all bladder tumors (2). These include small cell neuroendocrine carcinoma (SCNEC), large cell neuroendocrine carcinoma (LCNEC), well-differentiated neuroendocrine neoplasms, and paragangliomas (PGLs) (3). SCNEC and LCNEC are high-grade and clinically aggressive, whereas well-differentiated neuroendocrine neoplasms and PGLs are biologically distinct entities with variable malignant potential. The latter two subtypes generally have a slightly better prognosis than the former two. However, the overall prognosis of BNEN is poor, and most cases present with muscular invasion, lymph node metastasis, and distant metastasis (4). Due to the rarity of this disease and the lack of prospective studies, its pathogenesis and clinical management remain unclear. This study describes the clinical and pathological characteristics of nine patients with BNEN to improve understanding of this disease. In addition, a literature review was conducted to summarize the existing evidence and further enhance understanding of this disease.

## Methods

This retrospective study included patients with pathologically confirmed BNEN who were treated at our institution between January 2014 and June 2024. All pathological slides were independently reviewed by at least two pathologists and classified according to the WHO Classification of Urinary and Male Genital Tumors. The diagnosis was based on hematoxylin and eosin (H&E) morphology and confirmed by immunohistochemical staining for neuroendocrine markers, including synaptophysin, chromogranin A, and CD56, with classification performed in accordance with WHO criteria. Data were extracted from the electronic medical records, including patient demographic characteristics, clinical manifestations, diagnostic procedures, pathological findings, treatment modalities, and follow-up outcomes. Follow-up data were obtained from outpatient records, inpatient records, radiologic reports, and telephone follow-up when necessary. The data cutoff was June 2024. Overall survival (OS) was defined as the interval from the date of pathological diagnosis to death from any cause or to the last confirmed follow-up. Patients who were alive at the last follow-up were treated as right-censored observations. Survival and follow-up durations were rounded to the nearest whole month.

All analyses were descriptive. Categorical variables were summarized as numbers and percentages, and continuous variables were summarized as mean with range or median with range, as appropriate. Because this was a single-institution retrospective case series including only nine patients, no formal hypothesis testing, regression analysis, Kaplan–Meier comparison, or treatment-effect estimation was performed. Outcomes between patients with and without immune checkpoint inhibitor exposure were not statistically compared. Any apparent difference in OS/follow-up duration was interpreted as a descriptive observation rather than a statistically supported survival trend.

## Cases

## Results

The clinicopathological characteristics, treatments and follow-up outcomes, and immunohistochemical findings with serum NSE levels are summarized in **Tables 1**-**3**, respectively. A total of 9 patients with histologically confirmed BNEN were treated at our institution, including 7 men and 2 women, with a mean age of 61 years (range, 27–87 years). The maximum tumor diameter ranged from 1.5 to 5.5 cm. Painless gross hematuria was the most common presenting symptom. One patient also had lumbosacral pain, one case was incidentally detected during a routine physical examination, and two patients presented with lower urinary tract symptoms. Most patients had locally advanced disease at presentation. According to postoperative pathological TNM staging (pTNM), the tumors ranged from pT2b to pT4b, and some cases were accompanied by lymph node involvement. Bone metastasis was clinically suspected in 3 patients, including 1 in whom possible lung metastasis was also considered; another patient had prostatic invasion.

**Table 1:** Clinicopathological characteristics of the nine patients with bladder neuroendocrine neoplasms.

Case	Age	Sex	Tumor size (cm)	Symptoms	Pathological stage (pTNM)	Pathology	Notes
1	38	M	3.5×3×3	Hematuria + Lumbosacral pain	T4bN1M1b	High-grade urothelial carcinoma (HGUC) with neuroendocrine differentiation	Bone metastases suspected
2	78	M	4.5×3×2.5	Painless gross hematuria	T3aN0M0	Bladder-centered SCNEC admixed with adenocarcinoma	Incidental prostatic acinar adenocarcinoma, Gleason score 3 + 3/Grade Group 1, <1%; no prostatic neuroendocrine carcinoma
3	67	M	4.5×4	Painless gross hematuria	T3bN0Mx	SCNEC (90%) + HGUC (10%)	Bone metastases suspected
4	27	M	1.5×1	Hematuria + Dysuria	T4aN0M0	SCNEC	Prostate invasion
5	33	M	2×1.5	Physical examination	T2bN1Mx	PGL	-
6	87	M	4.5×3.5×2	Hematuria + Painful urination	T3aN0M0	LCNEC	LCNEC 100%
7	73	F	5×3.5×2.5	Painless gross hematuria	T2bN1Mx	LCNEC	Metastatic lymph node showed urothelial carcinoma component
8	77	F	4.5×3×2.5	Painless gross hematuria	T3aN0M0	LCNEC + HGUC + carcinoma *in situ* (CIS)	HG, CIS
9	69	M	5.5×4×4	Painless gross hematuria	T4aN0Mx	LCNEC + HGUC	HG, 95% LCNEC, 5% HG, Bone and lung metastasis suspected

**Table 2:** Treatments and follow-up outcomes.

Case	Surgical procedure	Neoadjuvant therapy	Bone scan	Urinary diversion protocol	Adjunctive treatment	Radiotherapy	Vital status at last follow-up	Follow-up/survival time, months
1	✓	-	✓	Ileal orthotopic bladder	-	-	Alive	7 months, censored
2	✓	-	-	Ureterostomy	EP + Tislelizumab	✓	Alive	29 months, censored
3	✓	-	✓	Ureterostomy	EP + Disitamab Vedotin	✓	Alive	18 months, censored
4	-	-	-	Ileal orthotopic bladder	GC + Toripalimab	-	Dead	23 months
5	✓	-	-	Ileal orthotopic bladder	-	-	Dead	22 months
6	✓	-	-	Ureterostomy	-	-	Dead	6 months
7	✓	-	-	Ureterostomy	GC + Toripalimab	-	Dead	23 months
8	✓	-	-	Ureterostomy	VMAT + Tislelizumab	✓	Dead	25 months
9	✓	-	✓	Ureterostomy	-	✓	Dead	4 months

**Table 3:** Immunohistochemical findings and serum NSE levels.

Case	P53	P63	CgA (chromogranin A)	Ki67 (%)	CD56	Syn (synaptophysin)	MSH6	MSH2	MLH1	Villin	NSE (ng/ml)
1	-	+	+	50	+	+	-	-	-	+	-
2	+	-	-	60	+	+	+	+	-	+	14.67
3	+	+	+	80	+	+	+	+	+	+	38.57
4	-	-	-	60	+	+	+	+	+	+	8.35
5	-	-	+	10	+	+	+	+	+	+	12.89
6	+	-	+	80	+	+	+	+	+	-	11.85
7	+	-	-	80	+	+	+	+	+	-	9.84
8	+	-	-	70	+	+	+	+	+	+	9.84
9	+	-	-	90	+	+	+	+	-	-	6.35

The pathological subtypes showed marked heterogeneity: 4 cases were predominantly composed of, or purely consisted of, LCNEC; 3 cases belonged to the SCNEC spectrum; and the remaining cases were high-grade carcinomas with neuroendocrine differentiation or other special histological types. Other pathological features included carcinoma *in situ* in 1 case and a urothelial carcinoma component in a metastatic lymph node in 1 case. Most patients underwent transurethral resection of bladder tumor before definitive local treatment. In this cohort, perioperative systemic treatment was mainly determined after final pathological and immunohistochemical confirmation, and postoperative chemotherapy, radiotherapy, immunotherapy, or other combination strategies were selected for some patients according to tumor stage, histological subtype, general condition, and treatment feasibility. Bone scintigraphy was performed in 3 patients because of suspected bone metastasis. Urinary diversion included orthotopic ileal neobladder in 3 patients and cutaneous ureterostomy in 6 patients.

At the data cutoff in June 2024, 3 patients were alive and 6 had died. Using OS defined as the interval from pathological diagnosis to death from any cause or last follow-up, the observed OS/follow-up duration ranged from 4 to 29 months. The median observed follow-up time for the whole cohort was 22 months. The 3 surviving patients were right-censored at 7, 18, and 29 months, respectively. The 6 patients who died had OS times of 4, 6, 22, 23, 23, and 25 months, respectively. Immunohistochemical analysis showed that all cases were positive for CD56 and Syn. The Ki-67 index ranged from 10% to 90%, indicating high proliferative activity in most tumors. P53 was positive in most cases, P63 was positive in only a minority of cases, and CgA expression was variable. In cases with available records, peripheral blood neuron-specific enolase (NSE) levels ranged from 8.35 to 38.57 ng/ml.

Additional retrievable clinical information is summarized in **Table 4**. Documented smoking history was present in two patients. Relevant past history included previous partial cystectomy for bladder cancer, previous open surgery for bladder stone, previous repair for gastric perforation, and possible left ureteral injury. Preoperative urinary tract infection was documented in four patients, with positive cultures for Escherichia coli and Klebsiella pneumoniae in two cases and negative cultures in the remaining two cases. Renal or urinary findings included unilateral or bilateral renal cysts and polycystic kidney disease.

**Table 4:** Additional clinical variables and treatment-selection information of the nine patients.

Case	Presenting symptoms	Smoking history	Past history/comorbidity	Family history/previous urothelial carcinoma	Concomitant bladder stone	Preoperative urinary tract infection/urine culture	Renal findings
1	Hematuria; lumbosacral pain	✓	Partial cystectomy for bladder cancer 14 years earlier	-	-	-	-
2	Painless gross hematuria	-	-	-	-	-	Bilateral renal cysts
3	Painless gross hematuria	-	-	-	-	-	Left renal cyst
4	Painless gross hematuria; dysuria	-	-	-	-	Yes/Escherichia coli	Right renal cyst
5	Incidentally detected by physical examination	✓	-	-	-	-	Right renal cyst
6	Dysuria; gross hematuria	-	Previous open surgery for bladder stone	-	-	Yes/Negative	Polycystic kidney disease
7	Painless gross hematuria	-	-	-	-	Yes/Negative	Left renal cyst
8	Painless gross hematuria	-	-	-	-	Yes/Klebsiella pneumoniae	-
9	Painless gross hematuria	-	Previous repair for gastric perforation; possible left ureteral injury	-	-	Yes/Negative	-

## Discussion

Neuroendocrine tumors (NETs) are a group of malignant neoplasms arising from neuroendocrine cells, some of which are capable of secreting hormones or bioactive peptides (5). Neuroendocrine cells and neuroendocrine neoplasms are widely distributed across multiple organ systems in the human body. The most common primary site is the gastrointestinal tract, followed by the lung, while the pancreas is also a frequent site of origin (6). BNEN is rare, accounting for less than 1% of all primary bladder cancers. This rarity is mainly attributable to the extremely small number of neuroendocrine cells in the bladder. As part of the urinary tract, the bladder is composed predominantly of urothelium (transitional epithelium), whereas neuroendocrine cells are present only in limited locations, where they mainly serve local regulatory rather than endocrine functions (7). Nevertheless, BNEN retains the typical immunophenotype of neuroendocrine neoplasms, with high expression of markers such as NSE, synaptophysin (Syn), and chromogranin A (CgA). These features suggest that BNEN shares substantial similarities with high-grade neuroendocrine neoplasms arising in the lung and other sites in terms of pathology, biological behavior, and treatment patterns. In addition, LCNEC is even rarer, and the development of optimal treatment strategies for this subtype is more challenging (8, 9). Primary bladder paraganglioma (PGL) is also exceedingly rare, accounting for less than 0.6% of all bladder tumors. This tumor is classified as an extra-adrenal pheochromocytoma and accounts for approximately 18% of all pheochromocytomas (10). Compared with SCNEC and LCNEC, PGL has an even lower incidence. Previous studies have shown that patients with SCNEC and LCNEC generally have worse overall survival (OS) and cancer-specific survival (CSS) than those with bladder urothelial carcinoma, adenocarcinoma, or PGL (11).

Most patients present with hematuria as the initial symptom, while some lesions are incidentally detected during biopsy or initial transurethral resection of bladder tumor (TURBT). Due to the extreme rarity of primary bladder paraganglioma (PGL) and LCNEC, current imaging knowledge is largely derived from studies on SCNEC (12). Compared with urothelial carcinoma (UC), bladder SCNEC typically presents with larger tumor size, lower apparent diffusion coefficient (ADC) values, and slightly higher CT attenuation, suggesting higher cellular density and greater aggressiveness (12–14). LCNEC generally appears as a non-specific mass on imaging (15), whereas bladder PGL typically presents as a hypervascular lesion with characteristic signal changes and restricted diffusion (16). However, a definitive diagnosis remains difficult based on imaging findings alone.

**Figure 1:**
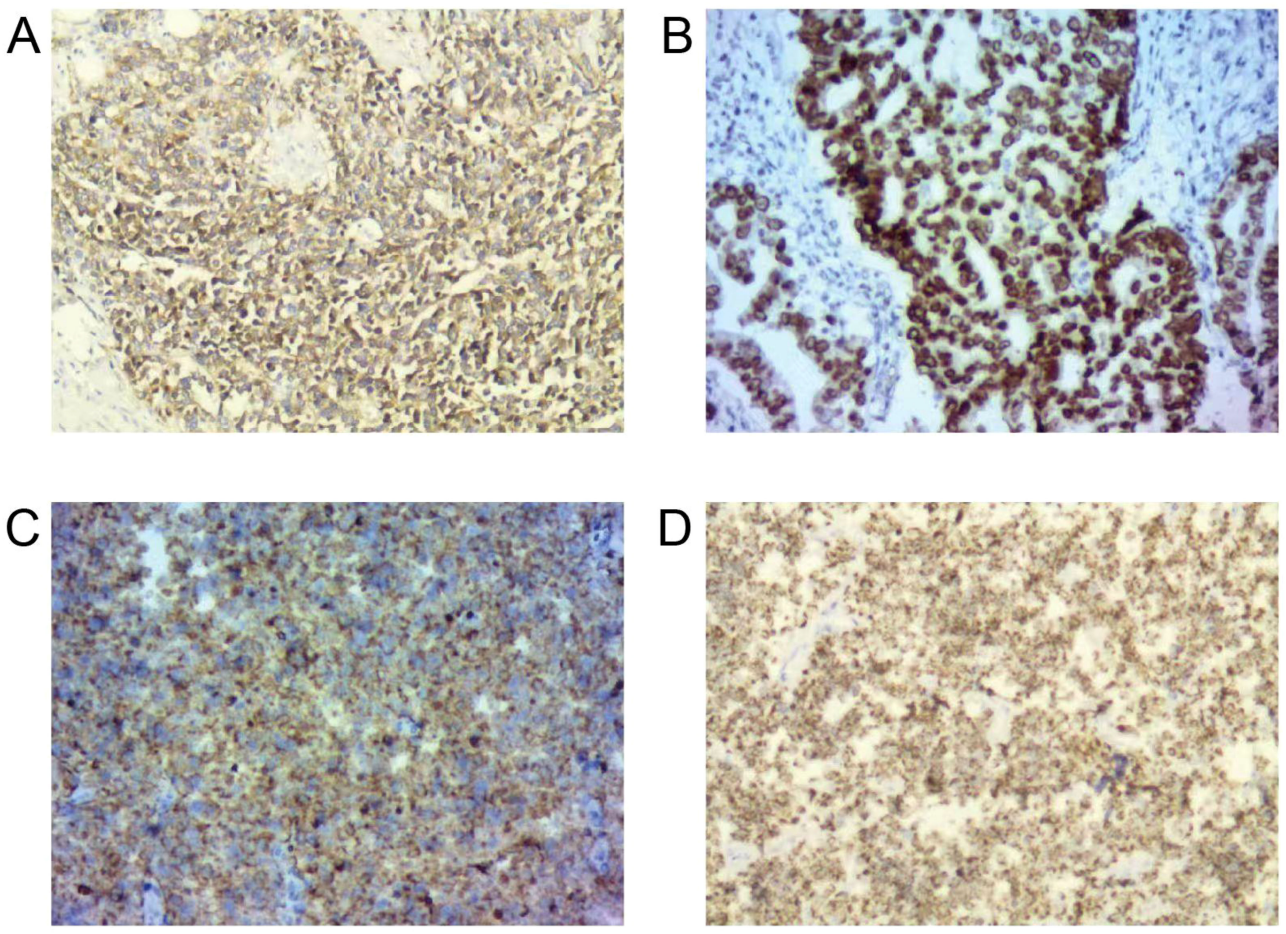
Representative immunohistochemical findings in bladder neuroendocrine neoplasms. **(A)** Synaptophysin (Syn) expression in a small-cell neuroendocrine carcinoma (SCNEC) case, supporting neuroendocrine differentiation. **(B)** Nuclear GATA3 staining in a mixed tumor containing small-cell neuroendocrine carcinoma and high-grade urothelial carcinoma components. GATA3 was interpreted together with morphology and the remaining immunohistochemical panel, rather than as an isolated neuroendocrine marker. **(C)** Chromogranin A (CgA) positivity in the mixed SCNEC/high-grade urothelial carcinoma case, illustrating neuroendocrine marker expression that may be present but is not uniformly expressed across all cases. **(D)** CK7 expression in a large-cell neuroendocrine carcinoma (LCNEC) case, indicating epithelial differentiation; CK7 was not regarded as a neuroendocrine-specific marker. These archived immunohistochemical photomicrographs are provided to illustrate qualitative staining patterns.

Therefore, the diagnosis should rely on the integrated assessment of histological morphology and immunohistochemical findings. Neuroendocrine markers such as CD56, synaptophysin (Syn), and chromogranin A (CgA) are frequently positively expressed and help distinguish BNEN from urothelial carcinoma (17, 18). Histologically, LCNEC is composed of relatively large cells with marked atypia and abundant cytoplasm, whereas SCNEC is characterized by smaller cells with scant cytoplasm and inconspicuous nucleoli (17, 18). In addition, markers such as GATA3, CD117, and TTF-1 may further improve diagnostic accuracy (19, 20). Immunohistochemical analysis of the 9 cases showed that CD56 was positive in all cases, while the positive rates for Syn and CgA were 88.9% and 44.4%, respectively. The Ki-67 index ranged from 10% to 90%, and most cases showed high proliferative activity, supporting neuroendocrine differentiation. Representative images showed positive staining for Syn and CgA, CK7 expression in some cases, and nuclear positivity for GATA3 (**Figure 1**).

**Figure 2:**
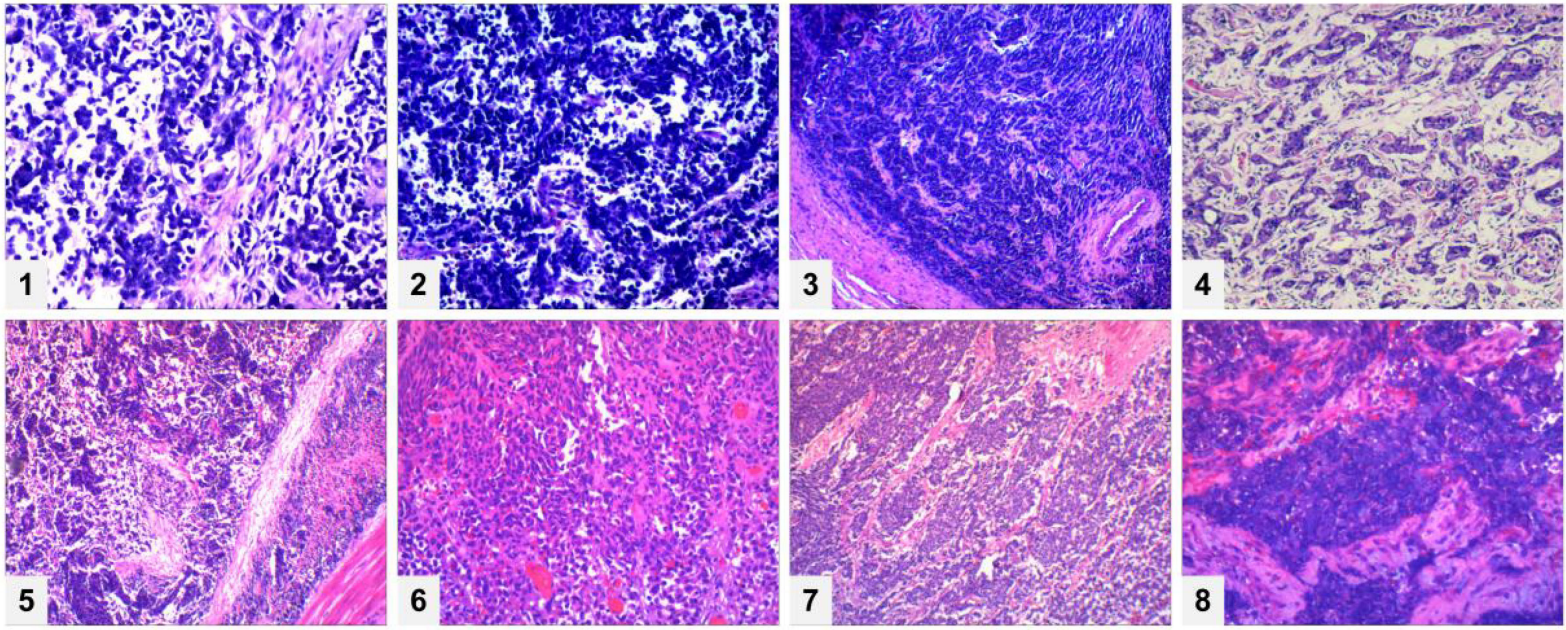
Representative hematoxylin and eosin (H&E)-stained histopathological features of bladder neuroendocrine neoplasms. (1) Small-cell neuroendocrine carcinoma (SCNEC) characterized by densely arranged small tumor cells with scant cytoplasm, hyperchromatic nuclei, and a high nuclear-to-cytoplasmic ratio. (2) Mixed carcinoma with a predominant SCNEC component, showing compact sheets of small hyperchromatic tumor cells; focal necrotic change is present where recognizable. (3) Mucosal SCNEC showing diffuse proliferation of small round-to-oval tumor cells with nuclear crowding and limited cytoplasm. (4) Well-differentiated neuroendocrine tumor, grade 2 (NET G2), showing relatively uniform tumor cells with an organoid/nested growth pattern. (5) Large-cell neuroendocrine carcinoma (LCNEC) showing larger atypical tumor cells with relatively abundant cytoplasm and solid or nested architecture. (6) LCNEC with marked cytological atypia and infiltrative growth, consistent with a high-grade neuroendocrine carcinoma. (7) LCNEC associated with high-grade urothelial carcinoma and carcinoma *in situ*, showing heterogeneous tumor architecture with solid and trabecular growth patterns. (8) Poorly differentiated carcinoma with neuroendocrine morphology, favoring SCNEC, showing diffuse sheets of hyperchromatic small tumor cells.

In addition, the histological composition of this cohort was complex, including not only pure small cell or LCNEC, but also mixed tumors containing components of high-grade urothelial carcinoma, adenocarcinoma, or carcinoma *in situ*. These findings suggest that BNEN does not arise through a single pathogenic pathway but instead exhibits heterogeneity similar to that of high-grade neuroendocrine neoplasms in other organs. This heterogeneity was also reflected in the representative pathological images, not only in differences among diagnostic subtypes but also in the morphological features observed on hematoxylin and eosin staining. Specifically, some tumors were composed of densely packed small cells with hyperchromatic nuclei and a high nuclear-to-cytoplasmic ratio, consistent with the morphology of SCNEC; in contrast, other tumors consisted of larger cells with relatively abundant cytoplasm arranged in nests, trabeculae, or solid sheets, which more closely resembled the morphology of LCNEC. In some cases, organoid nesting patterns and variable degrees of stromal separation were also observed, indicating additional diversity in tissue architecture (**Figure 2**). Taken together, these results indicate that BNEN is not a single histological entity, but rather a spectrum of tumors sharing a common background of neuroendocrine differentiation while showing marked heterogeneity in cytological features, growth patterns, and accompanying histological components.

In terms of treatment, due to the rarity of this disease, there is currently no standardized therapeutic regimen, and clinical practice largely relies on experience from other high-grade neuroendocrine neoplasms, particularly small cell lung cancer (21, 22). For localized or locally advanced bladder small cell or neuroendocrine carcinoma, platinum-based multimodal therapy is generally favored, and neoadjuvant platinum–etoposide chemotherapy followed by radical cystectomy may represent an ideal treatment sequence for surgically fit patients when the diagnosis is established before definitive surgery (23, 24). This strategy is supported by retrospective data showing the chemosensitivity of non-metastatic small cell carcinoma of the bladder and favorable long-term outcomes among patients who respond to neoadjuvant chemotherapy (25). However, the translation of this ideal treatment pathway into routine practice is often constrained by several real-world factors. First, bladder neuroendocrine carcinoma is frequently mixed with urothelial carcinoma, adenocarcinoma, squamous differentiation, or carcinoma *in situ*, and the neuroendocrine component may be underestimated or not fully recognized in limited TURBT specimens. Definitive classification may therefore depend on extensive sampling and immunohistochemistry after radical surgery. Second, many patients present with severe hematuria, anemia, irritative urinary symptoms, urinary obstruction, pain, or suspected locally advanced disease, making timely local control clinically important. Third, advanced age, poor performance status, renal dysfunction, comorbidities, and limited tolerance of cisplatin-based chemotherapy may reduce the feasibility of several cycles of preoperative systemic therapy. Finally, patient preference, access to treatment, repeated hospital visits, and the prolonged interval required for neoadjuvant therapy may further influence treatment selection. Therefore, perioperative treatment patterns in retrospective cohorts should be interpreted within the context of real-world diagnostic uncertainty, symptom burden, and individualized decision-making. Recent multi-institutional studies of urinary neuroendocrine neoplasms also indicate that treatment patterns remain heterogeneous in the modern era because of the rarity and biological diversity of these tumors (26). Currently, PD-1/PD-L1 inhibitors are considered standard therapies for first-line or subsequent treatment of urothelial carcinoma (UC), but their application in other tumor types remains limited. However, preliminary data from the United States suggest that although PD-L1 expression is relatively low in LCNEC, the response rate to immune checkpoint inhibitors (ICIs) may be higher than expected. The efficacy of ICIs in PGL and SCNEC still requires further investigation (27, 28). In addition, Lohrisch et al. investigated the role of radiotherapy in local disease control of SCNEC and reported an estimated 3-year local recurrence rate of 60% (29). All patients in this cohort underwent radical cystectomy, and some received additional postoperative chemotherapy, radiotherapy, and immunotherapy, suggesting that multimodal therapy has become the main clinical strategy.

In this cohort, patients who received immune checkpoint inhibitor–based combination therapy appeared to have relatively longer survival. Among the 9 patients, 4 (Cases 2, 4, 7, and 8) received postoperative multimodal therapy including immune checkpoint inhibitors. The specific regimens included etoposide plus cisplatin (EP) combined with tislelizumab in 1 patient, gemcitabine plus cisplatin (GC) combined with toripalimab in 2 patients, and volumetric modulated arc therapy (VMAT) combined with tislelizumab in 1 patient. Among the 9 patients, 4 received postoperative multimodal treatment containing immune checkpoint inhibitors. Their observed OS/follow-up times were 23, 23, 25, and 29 months, whereas the corresponding times among patients without immune checkpoint inhibitors were 4, 6, 7, 18, and 22 months. However, this observation should be interpreted with caution because of the small sample size, retrospective design, and treatment heterogeneity. Given the highly aggressive nature of these tumors, the high risk of recurrence after conventional treatment, and the presence of urothelial components in some cases, immunotherapy, particularly when combined with chemotherapy or radiotherapy, may warrant further investigation in selected patients. Although immune checkpoint inhibitors have become an important component of treatment for advanced urothelial carcinoma and several case reports have described durable responses in small cell bladder carcinoma treated with pembrolizumab- or atezolizumab-based regimens, the evidence for immunotherapy in extrapulmonary high-grade neuroendocrine carcinoma remains inconsistent (30). For example, recent studies in high-grade extrapulmonary neuroendocrine carcinoma did not demonstrate a clear additional benefit from adding checkpoint inhibitors to first-line cytotoxic chemotherapy in unselected patients, and pembrolizumab-based treatment has shown limited efficacy in advanced progressive extrapulmonary poorly differentiated neuroendocrine carcinoma (31). Thus, the relatively longer survival observed in several patients receiving immunotherapy-based combination therapy in the present cohort should be interpreted as hypothesis-generating. This finding may reflect not only the effect of immune checkpoint blockade but also differences in tumor burden, histological components, postoperative treatment intensity, radiotherapy, chemotherapy, antibody–drug conjugate exposure, and patient selection. Future studies should incorporate PD-L1 expression, tumor mutational burden, mismatch-repair status, DLL3 expression, and transcription factor-defined molecular subtypes to identify patients who may benefit from immunotherapy-based strategies.

Recent molecular studies have further demonstrated that bladder small cell/neuroendocrine carcinoma is biologically heterogeneous. Differential expression of ASCL1, NEUROD1, and POU2F3 can define distinct molecular subsets of bladder small cell/neuroendocrine carcinoma, while PLCG2 or POU2F3 expression has been associated with shorter recurrence-free and overall survival in patients without metastatic disease undergoing radical cystectomy. DLL3 expression is also frequently observed in bladder neuroendocrine carcinoma and may represent a potential therapeutic target. These findings suggest that BNEN should not be regarded as a single pathological entity, but rather as a spectrum of tumors with overlapping morphology, divergent differentiation, and potentially different therapeutic vulnerabilities (32). Therefore, the present findings should be interpreted as descriptive clinicopathological observations rather than pooled prognostic or treatment-efficacy evidence.

BNEN is a rare, heterogeneous, and highly aggressive malignancy with a generally poor prognosis. This study delineates the basic clinicopathological features of BNEN, including its tendency for advanced-stage presentation at diagnosis and consistent neuroendocrine differentiation. Beyond these descriptive findings, the practical value of this case series is the integrated presentation of subtype heterogeneity, mixed pathological components, diagnostic immunohistochemistry, suspected metastatic evaluation, urinary diversion, postoperative treatment selection, and censored follow-up, which may assist individualized decision-making in routine clinical practice.

## Conclusion

BNEN is a rare, heterogeneous, and highly aggressive malignancy with a generally poor prognosis. This study delineates the basic clinicopathological features of BNEN, including its tendency for advanced-stage presentation at diagnosis and consistent neuroendocrine differentiation.

Our findings suggest that accurate pathological classification and individualized multimodal treatment are important for treatment planning. Future large-scale multicenter studies and molecular investigations are needed to deepen the understanding of its pathogenesis and to develop more effective, standardized therapeutic strategies for these rare bladder cancers.

## Data Availability

The raw data supporting the conclusions of this article will be made available by the authors, without undue reservation.
